# *PIK3CA *mutations are frequently observed in BRCAX but not *BRCA2* -associated male breast cancer

**DOI:** 10.1186/bcr3463

**Published:** 2013-08-23

**Authors:** Siddhartha Deb, Hongdo Do, David Byrne, Nicholas Jene, kConFab Investigators, Alexander Dobrovic, Stephen B Fox

**Affiliations:** 1 Department of Pathology, Peter MacCallum Cancer Centre, East Melbourne, VIC 3002, Australia; 2 Department of Pathology and the Sir Peter MacCallum Department of Oncology, University of Melbourne, Parkville, VIC 3052, Australia; 3 Kathleen Cuningham Foundation Consortium for research into Familial Breast Cancer, Peter MacCallum Cancer Centre, East Melbourne, VIC 3002, Australia

**Keywords:** *PIK3CA*, E547K, mTOR, familial, male breast cancer, *BRCA2*, BRCAX

## Abstract

**Introduction:**

Although a substantial proportion of male breast cancers (MBCs) are hereditary, the molecular pathways that are activated are unknown. We therefore examined the frequency and clinicopathological associations of the PIK3CA/mammalian target of rapamycin (mTOR) and mitogen-activated protein kinase (MAPK) pathways and their regulatory genes in familial MBC.

**Methods:**

High resolution melting analysis and confirmatory sequencing was used to determine the presence of somatic mutations in *PIK3CA *(exon 9 and 20), *AKT1 *(exon 4), *KRAS *(exon 2) and *BRAF *(exon 15) genes in 57 familial MBCs. Further analysis of the PIK3CA/mTOR pathway was performed using immunohistochemistry for the pAKT1, pS6 and p4EBP1 biomarkers.

**Results:**

*PIK3CA *somatic mutations were identified in 10.5% (6 of 57) of cases; there were no *AKT1*, *KRAS *or *BRAF *somatic mutations. *PIK3CA *mutations were significantly more frequent in cancers from BRCAX patients (17.2%, 5/29) than *BRCA2 *(0%, 0/25) carriers (*P *= 0.030). Two BRCAX patients had an E547K mutation which has only been reported in one female breast cancer previously. *PIK3CA *mutation was significantly correlated with positive pS6 (83.3% vs. 32.0%, *P *= 0.024) and negative p4EBP1 (100% vs. 38.0%, *P *= 0.006) expression, but not pAKT expression. Expression of nuclear p4EBP1 correlated with BRCA2 mutation carrier status (68.0% vs. 38.7%, *P *= 0.035).

**Conclusions:**

Somatic *PIK3CA *mutation is present in familial male breast cancer but absent in *BRCA2 *carriers. The presence of two of the extremely rare E547K *PIK3CA *mutations in our cohort may have specific relevance in MBCs. Further study of *PIK3CA *in MBCs, and in particular BRCAX patients, may contribute to further establishing the relevance of specific *PIK3CA *mutations in MBC aetiology and in the identification of particular patient groups most likely to benefit from therapeutic targeting with the novel *PIK3CA *inhibitors that are currently in development.

## Introduction

Recent studies characterising male breast cancer (MBC) show that these rare tumours are very different to their female counterparts [1,2]. In particular, there are notable distinctions between familial female and MBC with a different pattern of penetrance and genotypic phenotypic correlation in *BRCA1*, *BRCA2 *and BRCAX subsets [1]. While it is likely that hormonal influence is a significant contributor, as yet, the characterisation of oncogenic drivers by mutation analysis of even the most common gene mutations in MBCs has not been undertaken.

Several significant targetable oncogenes are known and relatively well described in female breast cancer (FBC). The most frequent gain of function mutations is seen in phosphatidylinositol-4,5-bisphosphate 3-kinase, catalytic subunit alpha 9 *(PIK3CA) *which forms one of the catalytic subunits of the phosphatidylinositol 3-kinase (PI3K) holoenzyme [3,4]. Mutations of the helical or kinase domain lead to activation of the p110a kinase with subsequent downstream triggering of the mammalian target of rapamycin (mTOR) leading to cell proliferation, angiogenesis and promotion of the metastatic process [5,6]. Additional regulators of the PIK3CA/mTOR pathway include *AKT1 *and the RAS/RAF/mitogen-activated protein kinase (MAPK) pathway that intersect at multiple points [7-13].

Within FBC, the prevalence and prognostic significance of tumours with these driving mutations are unclear and may be dependent on both tumour histological type and estrogen receptor (ERα) status [14-17]. Notably, *in vitro *studies propose that activation of the PIK3CA/mTOR pathway may be important in tumours with deficient homologous recombination [18], suggesting a possible role in gaining resistance to poly ADP ribose polymerase (PARP) inhibitors in *BRCA1*/2 deficient tumours. However, although there are limited data (*n *= 22), an association between *BRCA1*/2 loss and activation of the PIK3CA/mTOR pathway in human tumours has not been confirmed [15].

Despite accruing data in FBC as to the significance of these oncogenes, there are few studies examining somatic mutation in sporadic MBC only [19-23], with the majority of studies focused on gene expression profiling [24-26] and germ-line mutational analysis [27-32].

Since the PIK3CA/mTOR pathway is more frequently associated with ERα positive FBC, and MBC is largely characterised by ERα positive disease, we have examined the frequency of activation of the PIK3CA/mTOR pathway and its regulators in a cohort of 57 familial MBCs. While the reported frequency of *KRAS *and *BRAF *mutations in female breast cancer is generally low (<5%) reference [33,34], a single sporadic MBC study showing a markedly high percentage of *KRAS *mutations (12%) also encouraged investigation of the mitogen-activated protein kinase (MAPK) pathway, which also interacts with the PIK3CA/mTOR pathway. Our aims were to; (1) identify *PIK3CA, AKT1, KRAS *and *BRAF *mutations in familial male breast cancer, (2) assess the relationship between such somatic gene mutations and clinicopathological factors, including *BRCA1/2 *mutation carrier status, and (3) identify and characterise the PIK3CA/mTOR and MAPK pathway and correlate with any clinicopathological factors and survival.

## Materials and methods

### Patient samples

Only primary breast cancers were examined in this study. Cases were obtained from the kConFab repository [35]. Prerequisites for cases to be included into kConFab are a strong family history of breast and ovarian cancer (Breast and Ovarian Analysis of Disease Incidence and Carrier Estimation Algorithm (BOADICEA) scores [36] generated from family pedigree and stratified by *BRCA1/2 *mutation carrier status included as Additional file 1: Supplementary figure 1) with criteria for admission to the kConFab study as outlined previously [37]. Cases were from Australia and New Zealand and diagnosed between 1980 and 2009.

The flow of patients through the study according to the REMARK criteria [38] is listed in Additional file 2: Supplementary table 1. Of the 118 cases within the kConFab registry, 58 cases were excluded due to unavailability of tissue. Of the 60 cases where tissue was available, 2 cases had insufficient tumour tissue for DNA extraction or for a core to be taken for assembly of a tissue microarray (TMA) and a further single case had an extremely low DNA yield and insufficient material for tissue microarray. Fifty seven cases had sufficient material at an appropriate DNA concentration for somatic mutation testing and one case did not have adequate tissue for TMA construction with all tissue committed to DNA extraction. Clinical parameters, including disease specific mortality were obtained from referring clinical centres, kConFab questionnaires and state death registries. Information on pedigree, mutational status and testing were available from the kConFab central registry. Histological classification was based on criteria set by the World Health Organization 2012 [39] and all slides and pathological records from all cases were reviewed for tumour size, tumour grade, lymphovascular and perineural invasion. Immunohistochemistry for ERα, progesterone receptor (PgR), basal markers (cytokeratin (CK) 5, epidermal growth factor receptor (EGFR)) and HER2 silver *in situ *hybridisation (SISH) had been performed as previously reported [1]. Using stratification of intrinsic phenotypes based on Nielsen *et al*. [40], tumours were placed into luminal (ERα positive, HER2 negative, CK5 and/or EGFR negative or positive), basal (HER2 and ERα negative; CK5 and/or EGFR positive), HER2 (HER2 positive, ERα, CK5 and EGFR negative or positive) and null/negative (HER2, ERα, CK5 and EGFR negative) phenotypes. This work was carried out with approval from the Peter MacCallum Cancer Centre Ethics Committee (Project No: 11/61). The approval included waiver of patient consent.

### Germline *BRCA1*/*2 *testing

Mutation testing for *BRCA1 *and *BRCA2 *mutations was performed as reported previously [1]. Testing of index cases in kConFab families was carried out by denaturing high performance liquid chromatography or multiplex ligation-dependent probe amplification. Once the family mutation had been identified, all pathogenic (including splice site) variants of *BRCA1 *and *BRCA2 *were genotyped by kConFab in all available family members' DNA.

### High-Resolution Melting (HRM) assay

Genomic DNA was extracted from formalin-fixed, paraffin embedded (FFPE) samples. A 3 μM haematoxylin and eosin (H&E) stained slide was cut from FFPE blocks and stained to identify tumour enriched areas. From the relevant area on the FFPE block, a 2 mm punch biopsy core was taken. The cores were then dewaxed and hydrated through gradient alcohol. Genomic DNA was then extracted using the DNeasy Tissue kit (Qiagen, Valencia, CA, USA)) following proteinase K digestion at 56°C for three days.

The *PIK3CA*, *AKT1*, *BRAF *and *KRAS *primer sequences are shown in Additional file 3: Supplementary table 2. *PIK3CA *exon 9 and 20 primers produced amplicons with 104 base pairs (bp) and 102 bp, respectively. *AKT1 *exon 4, *BRAF *exon 15 and *KRAS *exon 4 primers produced 78 bp, 144 bp and 92 bp amplicons, respectively. PCR for HRM analysis was performed in 0.1 ml tubes on a Rotor-Gene Q (Qiagen) utilising the fluorescent DNA intercalating dye, SYTO 9 (Invitrogen, Carlsbad, CA, USA). A 20 μL final reaction volume contained 1 × PCR buffer, 0.5 to 2.0 mM MgCl2, 200 to 400 nM of forward and reverse primer, 200 μM of dNTPs, 5 μM of SYTO 9, 0.5 U of HotStarTaq polymerase (Qiagen), 5 ng of genomic DNA, Uracil-DNA glycosylase (UDG) (0.5 units/reaction), UDG buffer (New England BioLabs, Ipswich, MA, USA) and PCR grade water. The cycling and melting conditions are shown in Additional file 3: Supplementary table 2. All reactions had initial UDG treatment for FFPE artefacts at 37°C for 30 minutes [41], followed by an incubation step at 95°C for 15 minutes, denaturation step at 95°C, annealing steps at the temperatures listed in Additional file 3: Supplementary table 2, and an elongation step at 72°C. A single cycle of 97°C for one minute preceded a melt phase run between temperatures listed in Additional file 3: Supplementary table 2 and rising 0.2°C per step. Samples were run in duplicate. HRM analysis was performed on the Rotor-Gene Q Software (v1.7) (Qiagen, Valencia, CA, USA).

### DNA sequencing

**Figure 1:**
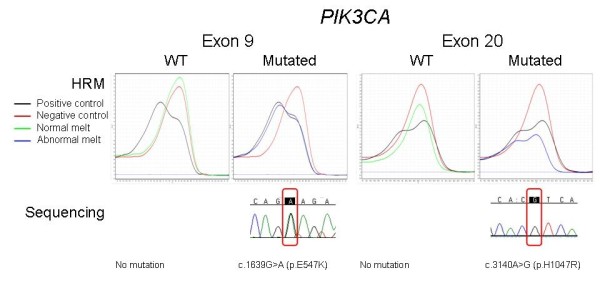
**Work flow of sample analysis**. Initial High-Resolution Melting (HRM) analysis was used to screen mutation by abnormal melt. Sanger sequencing was then performed to detect specific mutations.

All samples with either or both duplicates showing abnormal melt were sequenced for detection of mutations. *PIK3CA *exon 9 and 20 HRM products were amplified using M13 tagged primers (Additional file 3: Supplementary table 2) initially and then M13 primers for a second step for *PIK3CA *exon 9 (amplicon 185 bp) and a single step PCR reaction for *PIK3CA *exon 20 (amplicon 149 bp) using primers listed in Additional file 3: Supplementary table 2. The composition of a total reaction mixture of 20 μL contained; 1 × PCR buffer, 2.5 mM MgCl2, 400 nM of each primer, 200 μM of dNTPs, 0.5 U of HotStarTaq polymerase (Qiagen), 5 ng of HRM DNA products and PCR grade water. The PCR conditions were as follows: an initial incubation at 95°C for 1 minute, followed by 35 cycles of 95°C for 10 seconds, 55°C for 10 seconds and 72°C for 4 minutes. The sequencing reaction was then performed using the Big Dye Terminator v3.1 chemistry according to the manufacturer's protocol (Applied Biosystems, Foster City, CA, USA) using 6 μL of the PCR products that were purified with 2 μL of ExoSapIT (GE Healthcare, Little Chalfont, Buckinghamshire, UK). After ethanol precipitation, the sequencing products were run on a 3700 Genetic Analyser (Applied Biosystems). The sequencing data were then analysed using Sequencher 4.6 (Gene Codes Corporation, Ann Arbor, MI, USA). Each mutation was confirmed by sequencing a second independent PCR reaction. The work flow is outlined in Figure 1.

### Immunohistochemistry

Tumour-tissue microarrays (1-mm cores), with a two-fold redundancy, were prepared from archival FFPE tissue blocks. TMA sections were cut from each block at 4 μm thick intervals, dewaxed, placed through graded alcohol and then into water.

**Figure 2:**
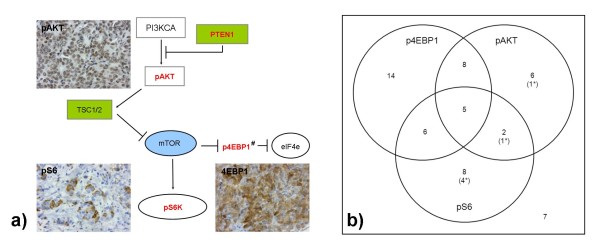
**PIK3CA/mTOR pathway (# 4EBP1 is inactivated by phosphorylation) a)**. Immunohistochemistry was performed for phosphorylated AKT, pS6 and p4EBP1. **b**) Outcome of immunohistochemical staining integrating *PIK3CA *mutation status. Numbers indicate the amount of cases showing positive immunohistochemical staining. Mutations are signified by (n*), with n = number of cases. mTOR, mammalian target of rapamycin.

For phosphorylated 4EBP1 (p4EBP1) and phosphorylated S6 (pS6), antigen retrieval was performed using high pH antigen retrieval buffer (DAKO, Glostrup, Denmark) in pressure cooker for three minutes at 125°C. For phosphorylated AKT1 (pAKT), antigen retrieval was performed with CC1 high pH retrieval solution (Roche, Basel, Switzerland) at 100°C for 36 minutes. Staining for p4EBP1 (dilution 1:400, clone 2855, Cell Signalling Technology, Danvers, MA, USA) and pS6 (dilution 1:200, clone 2211, Cell Signalling Technology) was performed using a monoclonal and polyclonal rabbit antibodies respectively. Antigen-antibody complex was detected using the Envision FLEX system (EnVision FLEX/HRP and EnVision FLEX DAB + Chromogen, DAKO). Staining for pAKT1 (dilution 1:1,000, clone LP18, Novocastra, Newcastle upon Tyne, UK) was performed using a monoclonal mouse antibody with secondary detection using Ventana Ultraview detection reagents (Roche). Slides were then counterstained with haematoxylin, dehydrated, cleared and mounted for assessment. Phosphorylated 4EBP1 expression was assessed for both cytoplasmic and nuclear expression, nuclear expression for pAKT1 and cytoplasmic expression for pS6 (Figure 2a). A histoscore was generated by multiplying staining intensity (0, no staining; 1, weak; 2, moderate; 3, strong) by the percentage of positive tumour cells (0, 0; 1, < or = to 25%; 2, >25% to 50%; 3, >50% to 75%, 4, >75%). The histoscores ranged between 0 and 12. For subsequent analysis, histoscores were categorised into either absent (histoscore = 0) or present (1 to 12) or low (0 to 3) and high (4 to 12) to differentiate from baseline staining of adjacent normal breast epithelium.

A *PIK3CA *mutation phenotype was defined by either a tumour harbouring a somatic *PIK3CA *activating mutation or showing an absence of p4EBP1 expression and moderate to strong pS6 expression (histoscore 4-12/12) on immunohistochemistry.

### Statistical analysis

Comparison of groups was made using Mann-Whitney U for non-parametric continuous distributions and chi-square test for threshold data. Kaplan-Meier survival curves were plotted using breast cancer related death as the endpoint and compared using a log rank test. Analysis was performed with GraphPad Prism 5 software (GraphPad Prism version 5.04 for Windows, GraphPad Software, La Jolla, CA, USA). A two-tailed *P*-value test was used in all analyses and a *P*-value of less than 0.05 was considered statistically significant.

## Results

### *PIK3CA *is commonly mutated in familial male breast cancer

Seven *PIK3CA *mutations were identified and confirmed in six samples (Table 1). Four activating mutations were identified in exon 9, with two cases of E547K mutation and one sample demonstrated concurrent E542K and E547K mutations in exon 9. Three further mutations were identified in exon 20, all of which were H1047R mutations. Screening of *AKT1*, *BRAF *and *KRAS *showed no evidence of somatic mutations.

**Table 1:** Somatic *PIK3CA *mutations in familial male breast cancer

Nucleotide change	Amino acid change	BRCA status
c.1624G>A	p.E542K	BRCAX
c.1639G>A	p.E547K	BRCAX
c.1624G>A, c.1639G>A	p.E542K, p.E547K	BRCAX
c.3140A>G	p.H1047R	BRCAX
c.3140A>G	p.H1047R	BRCAX
c.3140A>G	p.H1047R	*BRCA1 *del exons 21_24

### *PIK3CA *mutation is uncommonly seen in *BRCA2 *mutation carriers

**Figure 3:**
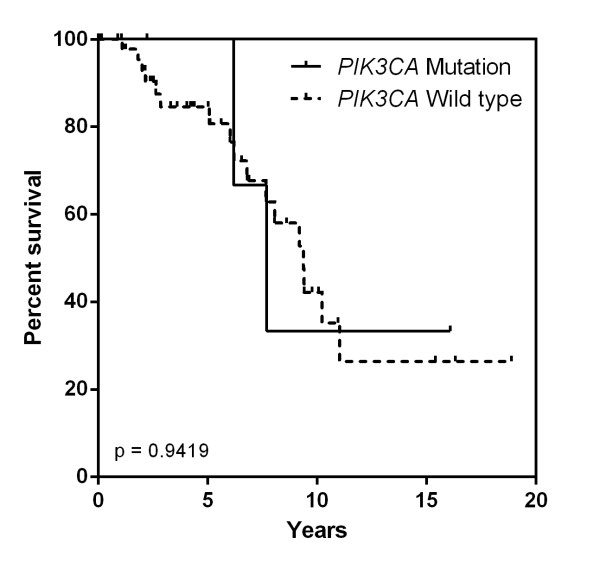
**Correlation of *PIK3CA *mutation and disease specific survival**.

One tumour arising in a *BRCA1 *carrier had an exon 20 *PIK3CA *mutation, five *PIK3CA *mutations occurred in BRCAX males whereas no *PIK3CA *mutation were identified in tumours from *BRCA2 *mutation carriers. There was a significant positive association between *PIK3CA *mutation incidence and BRCACX (17.2%) compared with *BRCA2 *(0%) associated tumours (*P *= 0.030). There was otherwise no correlation between the presence of somatic *PIK3CA *mutation and age of diagnosis, primary tumour size, tumour histological subtype, tumour grade, intrinsic phenotype, lymphovascular or perineural invasion (*P *> 0.05) (Table 2). The presence of *PIK3CA *mutation was not associated with a significant difference in Disease Specific Survival (DSS) (Figure 3).

**Table 2:** Correlation of *PIK3CA *mutation status with clinicopathological parameters

	*PIK3CA *Mutation (*n *= 6)	*PIK3CA *Wild type (*n *= 51)	*P*-value
**Age - mean (years)**	62.2	63.0	0.899
**Overall DSS**	33.3%	33.3%	1.000
**Carrier mutation status**			
*BRCA1*	1	2	0.288
*BRCA2*	0	25	**0.030**
BRCAX	5	24	0.194
**Primary tumour size (mm)**	18.8	19.1	0.948
**Histological type**			
Invasive carcinoma - no special type (IC - NST)	6	37	0.319
IC-NST with micropapillary areas	0	9	0.575
Invasive Papillary Carcinoma	0	3	1.000
Invasive Lobular Carcinoma	0	2	1.000
**Tumour grade**			
1	0	3	1.000
2	3	26	1.000
3	3	22	1.000
**Lymphovascular invasion**	33.3%	39.2%	1.000
**Perineural invasion**	50.0%	41.2%	0.689
**Intrinsic subtype**			
Luminal	6	45	1.000
HER2	0	5	1.000
Basal	0	1	1.000
Null	0	0	1.000

*P*-values < 0.05 in bold text.

### Co expression and clinicopathological correlation of p4EBP1, pS6, pAKT biomarkers

Cytoplasmic expression of p4EBP1 was present (histoscore 1 to 12) in 55.4% (31/56) of cases, nuclear p4EBP1 expression (histoscore 1 to 12) in 51.8% (29/56) of cases and either nuclear or cytoplasmic expression in 58.9% (33/56) of cases. High expression of both pS6 and pAKT1 (histoscore 4 to 12) was seen in 37.5% (21/56) of cases each. A pattern of co-expression of any of the markers was not seen (*P *> 0.05) (Figure 2b). Clinicopathological correlation showed that nuclear expression of p4EBP1 correlated with BRCA2 carrier status (17/25 (68.0%) *P *= 0.035) and inversely with BRCAX cases (11/30 (36.7%) *P *= 0.0184). There was no correlation between DSS and expression of any markers (Additional file 4: Supplementary figure 2).

### *PIK3CA *mutation phenotype

All tumours with *PIK3CA *mutation showed differences in some downstream pathway members. Expression of p4EBP1, pS6 and pAKT was observed in 0/6 (0%), 5/6 (83.5%) and 2/6 (33.3%) of cases respectively (Figure 2b). There was significant absence of p4EBP1 nuclear (*P *= 0.009) or cytoplasmic (*P *= 0.006) staining and up-regulation of pS6 (*P *= 0.024) in tumours with *PI3KCA *somatic mutations when compared with *PIK3CA *wild type (Table 3).

**Table 3:** Correlation of p4EBP1, pS6 and pAKT immunohistochemistry with *BRCA *status and clinicopathological factors

	4EBP1						pS6			pAKT		
	Cytoplasmic			Nuclear			Cytoplasmic			Nuclear		
	Present	Absent	*P*-value	Present	Absent	*P*-value	High	Low	*P*-value	High	Low	*P*-value
**PIK3CA Mutation**	**0**	**6**		**0**	**6**		**5**	**1**		2	4	
**PIK3CA Wild-Type**	**31**	**19**	**0.006**	**29**	**21**	**0.009**	**16**	**34**	**0.024**	19	31	1.000
												
**Carrier mutation status**												
BRCA1	1	2	0.581	1	2	0.605	1	2	1.000	0	3	0.284
BRCA2	15	10	0.596	**17**	**8**	**0.035**	9	16	0.582	10	15	0.786
BRCAX	15	13	0.456	**11**	**19**	**0.018**	11	17	1.000	11	17	1.000
												
**Age - mean (years)**	60.3	64.5	0.240	59.1	65.3	0.078	63.4	61.4	0.503	62.0	62.2	0.940
												
**Primary tumour size (mm)**	20.3	17.2	0.169	19.5	18.3	0.589	17.21	20.03	0.312	20.05	18.25	0.437
												
**Histological type**												
Invasive carcinoma - no special type (IC - NST)	21	19	0.563	22	19	0.766	13	28	0.212	15	26	1.000
IC-NST with micropapillary areas	6	3	0.716	3	6	0.288	6	3	0.066	3	6	1.000
Invasive Papillary Carcinoma	2	2	1.000	3	1	0.612	2	2	0.626	2	2	0.626
Invasive Lobular Carcinoma	2	1	1.000	1	1	1.000	0	2	0.523	1	1	1.000
												
**Tumour Grade**												
1	2	1	1.000	2	1	1.000	2	1	0.549	1	2	1.000
2	16	13	1.000	16	13	0.789	9	20	0.409	11	18	1.000
3	13	11	1.000	11	13	0.590	10	14	0.591	9	15	1.000
												
**LVI**	40.7%	36.0%	0.781	28.0%	48.2%	0.163	31.8%	43.3%	0.565	33.3%	41.9%	0.780
**PNI**	51.7%	45.8%	0.575	40.7%	57.6%	0.436	36.4%	58.1%	0.166	33.3%	59.0%	0.093
												
**Intrinsic subtype**												
Luminal	26	24	0.210	24	26	0.195	17	33	0.183	20	30	0.393
HER2	4	1	0.367	4	1	0.353	3	2	0.352	1	4	0.640
Basal	1	0	1.000	1	0	1.000	1	0	0.375	0	1	1.000
Null	0	0	1.000	0	0	1.000	0	0	1.000	0	0	1.000

## Discussion

This study is the first to characterise biomarkers and mutations in the PIK3CA/mTOR pathway in familial male breast cancer noting several novel observations. We identified a *PIK3CA *mutation rate of 10.5% in familial MBCs but an absence of common activating mutations of *AKT1*, *KRAS *and *BRAF*. While limited by moderate numbers in our study, the absence of *KRAS *mutation contrasts with the only other study performed in sporadic MBCs by Dawson *et al*. who reported an overall incidence of 12% [20]. Methodological reasons may be underlying these difference but in our experience, HRM is a highly sensitive and robust technique [42,43]. The absence of *BRAF *mutation is also somewhat expected and is supported by the stronger association between basal cell breast cancer lines and *BRAF *mutation [44] (since the majority of MBCs are of a luminal subtype). While a true frequency of these mutations requires further testing in a much larger cohort, these data suggest frequency is unlikely to be high and should parallel the range (0.7 to 5%) that is observed in female breast cancer.

The mutation rate of *PIK3CA *in this series is lower than the reported 17.9% (7/39) in the only other study performed, although this was in a population-based cohort of MBCs patients [19]. It is also less frequent than that reported in FBC (16.3% [19] to 40.0% [3]) (Table 4), which supports the notion that male breast cancer is biologically different from female breast cancer and that therapies that rely on the experience of the female disease are likely to be suboptimal. Furthermore, evidence from our data demonstrating that differences in this PIK3CA/mTOR pathway is dependent on the germline genotypes of male breast cancer, shows the basis of male breast cancer in *BRCA2 *mutation carriers is very different to that of BRCAX giving further credence to personalising breast cancer treatment whether male or female using individual patient and tumour characteristics. Thus, as the incidence of *PIK3CA *mutations in tumours from in *BRCA2 *carriers is likely to be negligible, these patients are unlikely to derive benefits from the PIK3CA inhibitors that are now entering clinical trials for female breast cancer [19].

**Table 4:** Comparison of *PIK3CA *mutation studies in male and female breast cancer

	Male Breast Cancer		Female Breast Cancer			
	Current Study	**Benvenuti S *et al*. **[20]	**Benvenuti S *et al*. **[20]	**Buttitta F *et al*. **[14]	**Campbell IG *et al*. **[3]	**Saal H *et al*. **[16]
**Study population**	High risk - familial	Population based	Population based	Population based	Population based	Population based
**Frequency**	6/57 (10.5%)	7/39 (17.9%)	14/86 (16.3%)	46/180 (25.6%)	28/70 (40.0%)	77/292 (26.4%)
**Mutation Locus**	3 exon 9, 3 exon 20	7 exon 20	6 exon 9, 8 exon 20	23 exon 9, 23 exon 20	15 exon 9, 9 exon 20, 3 exon 7, 1 exon 6	31 exon 9, 49 exon 20, 7 exon 7, 7 others
**Clinicopathological association**	Inverse correlation with *BRCA2 *mutation carrier status	No clinicopathological association	No clinicopathological association	Mutation seen more frequently in lobular carcinoma (46%, *P *< 0.001). Exon 9 more frequently seen in lobular carcinoma (30% of cases, *P *< 0.001).	No clinicopathological association	Association with ER positivity (*P *= 0.0001), PgR Positivity (0.0063) and lymph node positivity (*P *= 0.0375).

*P*-values < 0.05 in bold text

The distribution of mutations of *PIK3CA *in male breast cancer reported by Benvenuti *et al*. (Table 4) showed exclusively exon 20 mutations in MBC, supporting the suggestion that the frequency of exon 9 and 20 mutations may be gender and tissue specific. We, however, noted an equal distribution of exon 9 and 20 mutations, which is more reflective of the distribution seen by others in FBC [3,14]. Furthermore, the E547K mutation noted in two of our BRCAX patients has only once previously been reported in a single female breast cancer suggestive of a unique hot spot preferentially within male cancers. This mutation was detected and confirmed using HRM and Sanger sequencing in duplicate for each case using methodologies optimised for FFPE material. We have extensive experience with this methodology and feel it to be well suited and robust for formalin fixed paraffin embedded material. While we also acknowledge the occurrence of artifactual changes, the E547K mutation has not been detected in over 300 FFPE tumour samples we have screened to date (unpublished data) and thus, we feel that this mutation may be particular to a subset of MBC. The E547K mutation itself is found in the highly conserved helical domain of PIK3CA and possibly confers increased catalytic activity. The mutation is not unique to breast cancer, and has also been reported previously in one colorectal adenocarcinoma [45] and in seven neuroendocrine tumours of the lung [46] lending support for a true pathogenic mutation. Targeted sequencing of further MBC, and in particular non-BRCA2 tumours, may help determine a more accurate incidence and potential relevance of this uncommon mutation. We also observed a case with two concurrent exon 9 mutations, which has not been previously reported in MBC. While there is some suggestion of a more aggressive phenotype or of tumour heterogeneity in cases with dual *PIK3CA *mutations [16,47,48], the clinical significance of this is also unclear due to the infrequency of this observation.

Recent data show that *BRCA2 *appears to be a significant driver in MBC, with a considerably higher penetrance within male *BRCA2 *carriers compared with males in BRCAX families and *BRCA1 *male mutation carriers [1]. It is also noteworthy that *BRCA2 *somatic mutations have also been reported in 21.8% of sporadic MBCs [22]. Furthermore, unlike in FBC, studies by Ottini *et al*. [49] and ourselves [1] intimate a distinct *BRCA2 *phenotype in MBCs, which more commonly contain areas of micropapillary histology, are of a higher grade, are PgR negative and are HER2 amplified. The genomic findings of this study emphasize that *BRCA2 *tumours may be a distinct subgroup in familial MBC and as such *BRCA2 *mutation may be a significant driver in MBC. Further support for a strong inherent *BRCA2 *associated drive independent of gender and estrogenic influence in male breast cancer is the association of *PIK3CA *mutation and ERα positive female breast cancer [14-17], a phenotype which is common to *BRCA2 *associated male tumours (92%) [1], but without the associated rate of *PIK3CA *mutation. These data suggest that gender and hormonal dimorphism may not be so significant in *BRCA2 *carriers and that *BRC*A*2 *male breast cancers align with the non-*PIK3CA *mutated ERα positive group of female breast cancer.

*PIK3CA *oncogenic drive, however, may be more important in non-*BRCA2 *MBCs where estrogenic influences may be more prominent. While our previous studies have shown that ERα and PgR positive tumours were seen at a similar frequency across all *BRCA1*, *BRCA2 *and BRCAX cohorts and more commonly than in FBC [1], based on this genotypic analysis, the mechanism and effect of *PIK3CA *mutation is likely to be different between the subgroups. Overall, given the association between ERα positive tumours and increased *PIK3CA *mutation frequency in FBC, one would assume an increased rate of *PIK3CA *mutation in MBCs. This is not seen and may suggest alternate receptor and PIK3CA/mTOR interaction in male breast cancer or a dose-based relationship differentiated by male cancers with low estrogen at one end of the spectrum and higher levels of estrogen in females at the opposite end. While studies have extensively examined the correlation between hormone receptor status and incidence of *PIK3CA *mutation, as yet there are very limited data on the effect of circulating oestradiol on *PIK3CA *mutation rate with some suggestion that PIK3CA/mTOR activation may contribute to tamoxifen-resistance. Further evidence of estrogen influence is also provided by Benvenuti *et al*. who observed a gender bias for *PIK3CA *mutations in colorectal cancer with a higher incidence of mutations in women (23%) compared with men (9%) [19] (Table 4), which reflect the findings of our study. Further study correlating serum oestradiol, testosterone levels and *PIK3CA *mutation frequency in MBCs are required to further elaborate on a possible association.

Recent *in vitro *studies showing increased sensitization of cancers with defects in DNA homologous recombination (as seen in *BRCA1*/2 deficient cancers), to PARP inhibition by targeting of PIK3CA [18,50] suggest that PIK3CA/mTOR pathway interactions result in homologous recombination steady state. Support for the model is not yet seen *in vivo *with only one study to date to have examined a correlation between *BRCA *mutation carriers' status and *PIK3CA *mutation incidence in FBC. Limited by numbers, Michelucci *et al*. describe two mutations (one codon 9 and one codon 20) in 12 *BRCA2 *mutation carriers and no mutations in 10 *BRCA1 *mutation carriers [15]. The clinical value of this dual targeting is unknown in *BRCA1*/2 FBCs and whether it is male or female, this study is also the first to describe a *PIK3CA *somatic mutation in a *BRCA1 *mutation carrier. The low numbers of MBCs in *BRCA1 *mutation carriers in our study reflects the paucity of these tumours in this particular cohort, and in *BRCA1 *carriers in general [51-54]. What is apparent is that *BRCA1-*associated tumours in males appear to be more similar to the tumours seen in post-menopausal female *BRCA1 *carriers, with an absence of tumours arising in young patients and an absence of an association with basal cell phenotype. Notwithstanding, carrying a *BRCA1 *mutation does appears to be a risk factor for MBC with a higher incidence than that of the general population but at much lower penetrance than seen in female *BRCA1 *carriers and it is still unclear as to the role *BRCA1 *plays in MBC. While the findings in this study are novel, true incidence and relevance of *PIK3CA *mutations in this cohort require further investigation of larger numbers of *BRCA1 *patients, if these can be acquired for study.

The alignment of *PIK3CA *mutation with elevated pS6 expression and absent p4EBP1 expression is different to the expected model. Theoretically, *PIK3CA *mutational activation of the pathway should only lead to an elevated pS6, as is seen, but not an elevated p4EBP1 (the phosphorylated form being inactive) and pAKT, which is not observed. This is in part likely to be due to the complexity of the PIK3CA/mTOR pathway. Indeed, a correlation between *PIK3CA *mutation in luminal A FBC (the phenotype most similar to MBC) and combined up-regulation of pAKT, p4EBP1 and pS6 is not seen [55]. The association seen in the series between *PIK3CA *mutation and elevated pS6 (*P *= 0.024) may suggest partial activation of the PIK3CA/mTOR pathway in MBC and reflect the variability of pS6 and p4EBP1 and pAKT levels seen *in vitro *with dose dependent inhibition of mTORC1 [56], or interactions of mTORC2, other pathways and feedback loops.

Nevertheless, we observed up-regulation of p4EBP1 in *BRCA2 *mutation carriers (68.0%) more frequently than in BRCAX carriers (36.7%), an association not reported in FBC, giving further evidence to the difference in male and female breast cancers. It may be that an alternate mechanism of PIK3CA/mTOR pathway activation may be present in *BRCA2 *cases linked to disordered homologous recombination, as mentioned previously, through p4EBP1 and eIF4e.

## Conclusion

The results of this study indicate that somatic *PIK3CA *mutation are a frequent alteration in familial MBC of BRCAX families, the incidence and type of which is comparable to that seen in sporadic male and slightly lower than FBCs. Conversely, the absence of *PIK3CA *mutation in *BRCA2 *associated MBCs suggests that alternate oncogenic drivers minimally contribute to tumour drive in this group, thus supporting distinct male breast cancer types. The study has also revealed differences of MBC to FBC and between sporadic and familial MBC which are of importance in optimising treatment strategies and underlying relevance of the PIK3CA/mTOR pathway in tumour biology. Indeed, the therapeutic implications of these findings support the delineation of significant molecular pathways, such as PIK3CA/mTOR and MAPK cascades for subsequent targeted therapies within specific populations.

## Abbreviations

BOADICEA: Breast and Ovarian Analysis of Disease Incidence and Carrier Estimation Algorithm; Bp: base pairs; CK: cytokeratin; DSS: disease specific survival; EGFR: epidermal growth factor receptor; ERα: estrogen receptor alpha; FBC: female breast cancer; FFPE: formalin fixed paraffin embedded; H&E: haematoxylin and eosin; HRM: high resolution melt; IC-NST: invasive carcinoma of no special type; MAPK: mitogen activated pathway kinase; MBC: male breast cancer; mTOR: mammalian target of rapamycin; mTORC: mammalian target of rapamycin complex; pAKT: phosphorylated AKT; PARP: poly ADP ribose polymerase; PI3K: phosphatidylinositol 3-kinase; PIK3CA: phosphatidylinositol-4,5-bisphosphate 3-kinase, catalytic subunit alpha; PgR: progesterone receptor; pS6: phosphorylated S6; p4EBP1: phosphorylated 4EBP1; SISH: silver *in situ *hybridization; TMA: tissue microarray; UDG: uracil DNA glycosylase.

## Competing interests

The authors declare that they have no competing interests.

## Authors' contributions

AD performed the manuscript review, contributed to study concept and design and the HRM assay design. DB performed p4EBP1 and pS6 immunohistochemistry. HD developed HRM assays, and assisted in performing and interpreting Sanger sequencing. The kConFab Investigators performed germ-line *BRCA1/2 *testing on all patients, and acquired clinical data. NJ performed pAKT immunohistochemistry. SBF prepared the manuscript and contributed to study concept and design. SD performed HRM and Sanger Sequencing of samples, interpretation of pAKT, p4EBP1 and pS6 immunohistochemistry, statistical analysis and manuscript preparation. All authors read and approved the final manuscript.

## Supplementary Material

Additional file 1 **Supplementary figure 1: BOADICEA scores for patients included in study**. Probability (Prob) score (0 to 1) is generated for *BRCA1 *and *BRCA2 *mutations for each case, stratified by known BRCA status. Click here for file

Additional file 2 **Supplementary table 1**. REMARK criteria leading to cases recruitment. Click here for file

Additional file 3 **Supplementary table 2**. HRM and Sequence specific *PIK3CA, AKT1, KRAS *and *BRAF *primers. Click here for file

Additional file 4 **Supplementary figure 2**. Disease specific survival stratified by 1a) nuclear p4EBP1 expression, 1b) cytoplasmic p4EBP1 expression, 1c) pS6 expression and 1d) pAKT expression. Click here for file
