# Supplementary material for: PIK3CA mutations are frequently observed in BRCAX but not BRCA2 -associated male breast cancer
Source: Breast Cancer Res. 2013 Aug 23;15(4):R69. doi: 10.1186/bcr3463 (PMC3978692; doi:10.1186/bcr3463)
Supplement: Additional file 1 — Supplementary figure 1: BOADICEA scores for patients included in study. Probability (Prob) score (0 to 1) is generated for BRCA1 and BRCA2 mutations for each case, stratified by known BRCA status. [file bcr3463-S1.DOC]

|  | ***BRCA1*** | ***BRCA2*** | **BRCAX** | **Total** |
| --- | --- | --- | --- | --- |
| **1. Males present in kConFab registry** | 5 | 35 | 78 | 118 |
| **2. Patients with tissue available** | 3 | 25 | 32 | 60 |
| **3. Tumour tissue available for mutational analysis** | 3 | 25 | 29 | 57 |
| **4. Tumour tissue present on microarray** | 3 | 25 | 28 | 56 |
