# Supplementary material for: PIK3CA mutations are frequently observed in BRCAX but not BRCA2 -associated male breast cancer
Source: Breast Cancer Res. 2013 Aug 23;15(4):R69. doi: 10.1186/bcr3463 (PMC3978692; doi:10.1186/bcr3463)
Supplement: Additional file 2 — Supplementary table 1. REMARK criteria leading to cases recruitment. [file bcr3463-S2.DOC]

| **HRM Primers** | **Primer (Sequence 5' to 3')** | **Cycles** | **Annealing temperature** | **Melt** |
| --- | --- | --- | --- | --- |
| *AKT1* - exon 4 | AKT1 - HRM - exon 4 – Forward: CGAGGGTCTGACGGGTAGAGTG | 55 | 55^o^C | 70-95^o^C |
|  | AKT1 - HRM - exon 4 – Reverse: GGCCGCCAGGTCTTGATGT |  |  |  |
| *BRAF* - exon 15 | BRAF - HRM - exon 15 – Forward: CAGGAAACAGCTATGACCCATGAAGACCTCACAGTAAAAATAGGT | 60 | 55^o^C | 72-95^o^C |
|  | BRAF - HRM - exon 15 – Reverse: TGTAAAACGACGGCCAGTCATCCACAAAATGGATCCAGACAAC |  |  |  |
| *KRAS* exon 2 | KRAS - HRM - exon 2 – Forward: TTATAAGGCCTGCTGAAAATGACTGAA | 55 | 68^o^C | 70-90^o^C |
|  | KRAS - HRM - exon 2 – Reverse: TGAATTAGCTGTATCGTCAAGGCACT |  |  |  |
| *PIK3CA* - exon 9 | PIK3CA - HRM - exon 9 – Forward: AAAGAACAGCTCAAAGCAATTTCTACAC | 60 | 55^o^C | 70-90^o^C |
|  | PIK3CA - HRM - exon 9 – Reverse: TGCTGTTTAATTGTGTGGAAGATCC |  |  |  |
| *PIK3CA* – exon 20 | PIK3CA - HRM - exon 20 – Forward: TGAGCAAGAGGCTTTGGAGTATTTC | 55 | 55^o^C | 70-85^o^C |
|  | PIK3CA - HRM - exon 20 – Reverse: TGCTGTTTAATTGTGTGGAAGATCC |  |  |  |
| **Sequencing Primers** | **Primer (Sequence 5' to 3')** |  |  |  |
| *PIK3CA* – exon 9 | PIK3CA - exon 9 – Forward: TGTAAAACGACGGCCAGTCAGAGTAACAGACTAGCTAGAGACAATG |  | | |
|  | PIK3CA - exon 9 – Reverse: CAGGAAACAGCTATGACCAATCTCCATTTTAGCACTTACCTGTGAC |  |  |  |
| *PIK3CA* – exon 20 | PIK3CA - exon 20 – Forward: TCGACAGCATGCCAATCTCTTC |  | | |
|  | PIK3CA - exon 20 – Reverse: TGCTGTTTAATTGTGTGGAAGATCC |  |  |  |
| M13 Primers | M13 Forward: TGTAAAACGACGGCCAGT |  | | |
|  | M13 Reverse: TGTAAAACGACGGCCAGT |  |  |  |
