# Supplementary figures and images for: PIK3CA mutations are frequently observed in BRCAX but not BRCA2 -associated male breast cancer
Source: Breast Cancer Res. 2013 Aug 23;15(4):R69. doi: 10.1186/bcr3463 (PMC3978692; doi:10.1186/bcr3463)

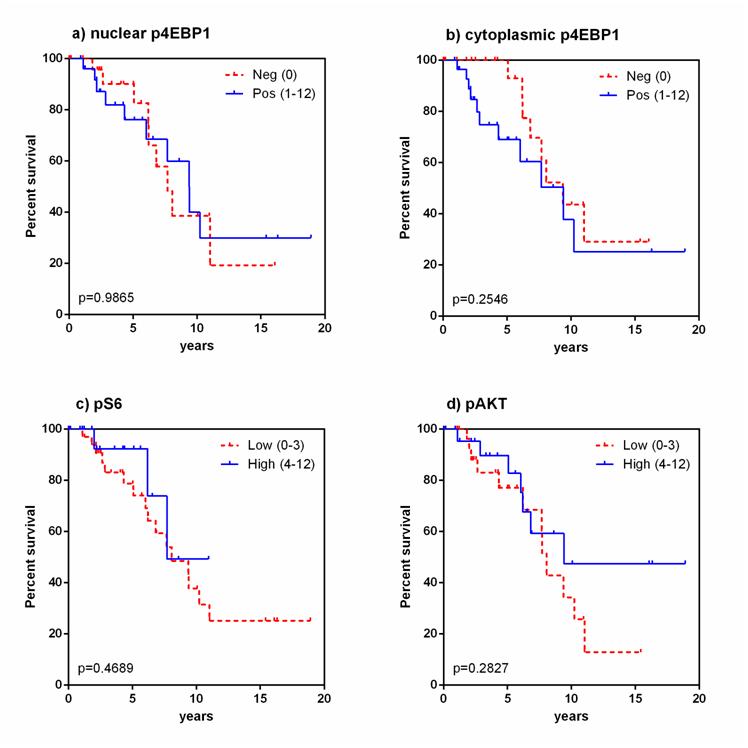

Supplement: Additional file 3 — Supplementary table 2. HRM and Sequence specific PIK3CA, AKT1, KRAS and BRAF primers. [file bcr3463-S3.JPEG]
